# Insecticide–impregnated dog collars reduce infantile clinical visceral leishmaniasis under operational conditions in NW Iran: A community–wide cluster randomised trial

**DOI:** 10.1371/journal.pntd.0007193

**Published:** 2019-03-04

**Authors:** Orin Courtenay, Ahad Bazmani, Parviz Parvizi, Paul D. Ready, Mary M. Cameron

**Affiliations:** 1 Zeeman Institute, and School of Life Sciences, University of Warwick, Coventry, United Kingdom; 2 Infectious and Tropical Diseases Research Centre, Tabriz University of Medical Sciences, Tabriz, Iran; 3 Pasteur Institute of Iran, Tehran, Iran; 4 London School of Hygiene and Tropical Medicine, Keppel Street, London, United Kingdom; University of Iowa, UNITED STATES

## Abstract

**Objective:**

To assess the effectiveness of community-wide deployment of insecticide–impregnated collars for dogs- the reservoir of *Leishmania infantum*–to reduce infantile clinical visceral leishmaniasis (VL).

**Methods:**

A pair matched–cluster randomised controlled trial involving 40 collared and 40 uncollared control villages (161 [95% C.L.s: 136, 187] children per cluster), was designed to detect a 55% reduction in 48 month confirmed VL case incidence. The intervention study was designed by the authors, but implemented by the Leishmaniasis Control Program in NW Iran, from 2002 to 2006.

**Results:**

The collars provided 50% (95% C.I. 17·8%–70·0%) protection against infantile VL incidence (0·95/1000/yr compared to 1·75/1000/yr). Reductions in incidence were observed across 76% (22/29) of collared villages compared to pair–matched control villages, with 31 fewer cases by the end of the trial period. In 11 paired villages, no further cases were recorded post–intervention, whereas in 7 collared villages there were 9 new clinical cases relative to controls. Over the trial period, 6,835 collars were fitted at the beginning of the 4 month sand fly season, of which 6.9% (95% C.I. 6.25%, 7.56%) were lost but rapidly replaced. Collar coverage (percent dogs collared) per village varied between 66% and 100%, with a mean annual coverage of 87% (95% C.I. 84·2, 89·0%). The variation in post-intervention clinical VL incidence was not associated with collar coverage, dog population size, implementation logistics, dog owner compliance, or other demographic variables tested. Larger reductions and greater persistence in incident case numbers (indicative of transmission) were observed in villages with higher pre-existing VL case incidence.

**Conclusion:**

Community–wide deployment of collars can provide a significant level of protection against infantile clinical VL, achieved in this study by the local VL Control Program, demonstrating attributes desirable of a sustainable public health program. The effectiveness is not dissimilar to the community-level protection provided against human and canine infection with *L*. *infantum*.

## Author summary

Zoonotic visceral leishmaniasis is a sand fly-borne disease of humans and dogs caused by the intracellular parasite *Leishmania infantum*. Dogs are the proven reservoir. The disease is of global health significance, and usually fatal unless treated. There are limited options to reduce transmission. Insecticide-treated dog collars have been shown to protect dogs against infectious bites from sand fly vectors, resulting in reductions of new infections in both dogs and humans. However, there have been no studies to demonstrate the public health benefits of this approach i.e. the impact on clinical VL incidence. This study assessed the effectiveness of community-wide deployment of insecticide–impregnated collars on dogs to reduce the incidence of clinical visceral leishmaniasis in children, the high risk age-group. Collars were fitted to dogs in 40 endemic villages over 4 consecutive years by the regional public health authorities in NW Iran. The case incidence of infantile visceral leishmaniasis in these villages was compared to that in 40 untreated villages at the end of the intervention period. The community-wide deployment of collars proved to provide a 50% reduction in the development of the disease in children. This effect was achieved under the operational conditions of the regional routine health authorities. We conclude that the implementation of insecticide-impregnated collars should be considered in strategic scale-up operations against zoonotic visceral leishmaniasis.

## Introduction

Visceral leishmaniasis (VL) is a protozoan vector–borne parasitic disease of humans following infection with *Leishmania donovani* or *L*. *infantum*, characterized by prolonged fever, wasting, splenomegaly, and hepatomegaly, and >95% case–fatality in the absence of treatment[1]. *Leishmania* are transmitted by female phlebotomine sand flies. Leishmaniasis is a Neglected Tropical Disease (NTD) strongly associated with poverty and malnutrition[2], resulting in a global incidence of 50,000 to 90,000 new VL cases per year[1]. Difficulties in reducing VL case burdens arise due to the current lack of a human vaccine, limited safe therapeutic drugs, need for improved vector control, and a better understanding of transmission dynamics[3–5].

VL due to *L*. *donovani* is anthroponotically transmitted, whereas VL due to *L*. *infantum* is a zoonosis involving infectious domestic dog reservoirs[6], and uncertain role of humans[7], in maintaining transmission[8]. Otherwise traditionally known as “infantile VL”, zoonotic VL is a disease mainly of young children[9–14], although case age-distributions may vary e.g. [15, 16].

Regional VL control programs focus on human case detection and treatment, adult sand fly vector control, and for dogs, optional strategies including canine vaccination, topical insecticide protection, chemotherapeutic treatment, or euthanasia[17–19]. Anthroponotic VL has been targeted for elimination as a public health problem (<1 case/10,000 people per year at district levels) in the Indian subcontinent by 2020, with substantial investment, technical and political support[3, 5, 20]. This has contributed to significant reductions in incidence between 2012 and 2017 [1, 12, 21]. In contrast, investment to reduce zoonotic VL in endemic Latin America, central Asia and Caucasia, are minimal by comparison; no such reductions in incidence are observed notably in the Americas where >90% of cases occur [22, 23].

Vector and animal reservoir control are necessary to reduce zoonotic VL incidence as treatment of human clinical cases, though necessary, is unlikely to impact on the transmission cycle. Insecticides topically applied to dogs as slow release insecticide–impregnated collars have been extensively tested in different regions, showing that they are effective against sand fly vectors[24–28], and reduce infection risk in dogs[29–37]. Evidence that collars also can reduce human infection incidence is limited to a single cluster randomized trial conducted in NW Iran[31]. However, there are no peer-reviewed studies to test the impact of this approach on human clinical VL disease.

In collaboration with the regional VL control program authorities in NW Iran, and with access to VL case records provided by the Ministry of Health (MoH), we had the opportunity to evaluate the impact of community–wide distribution of collars against clinical VL incidence, and as conducted under operational conditions. The principal aims of the study were (i) to measure the efficacy of the collar intervention against VL case incidence; (ii) to evaluate the operational logistics of collar implementation; and (iii) to assess likely causes for the variation in intervention effectiveness.

## Methods

### Study location

The study was conducted in the rural communities of the Kalaybar and Ahar administrative districts of East Azerbaijan province, NW Iran (38688131N; 4321696E) from 2002 to 2006. Villages were located at altitudes of 369-1305m (Table 1). The main economic activity in the area is agriculture, cash crops and particularly sheep farming. Dogs are kept as shepherd dogs or household guard dogs, and small numbers of livestock (sheep, goats, cattle, chickens) are kept in shelters variably near or attached to houses. Houses are constructed of plastered or unplastered stone, cement, or brick, and rooves constructed of cement, thatched or corrugated iron. Dried manure is generally stocked in piles away from houses as a source of fertilizer and fuel. Temperatures ranged from 1°C (in January) to 23°C (in July/August), and rainfall from 18mm (in August) to 76mm (in May).

**Table 1:** Demographic characteristics of trial villages (clusters).

	Collar arm	Control arm		
**Total numbers per arm**				
Villages	40	40		
Human population	22904	22844		
Children 0–10 yrs	6341	6562		
Households	3760	4011		
Dogs	1962	1670		
Houses with ≥1 dog	1364	1387		
**Geometric mean (95% C.L.s) per village**			Wilcoxon rank-sum z	P≤
Proportion of the population 0–10 yrs	0·27 (0·256, 0·283)	0·29 (0·270, 0·301)	1.29	0.190
M: F sex ratio children 0–15 yrs	0·99 (0·921, 0·106)	1·03 (0·978, 1·09)	0.85	0.394
Ratio shepherd: guard dog per village	0·45 (0·350, 0·589)	0·46 (0·344, 0·570)	0.55	0.389
Households per village	72 (56·4, 91·4)	82 (66·9, 100·8)	1.00	0.319
Humans population per village	469 (383·3, 574·2)	478 (392·9, 580·4)	0.20	0.840
Children 0–10 yrs per village	126 (101·1, 157·7)	136 (112·0, 165·6)	0.50	0.620
Dogs per village	43 (36·5, 50·2)	37 (31·5, 43·6)	0.69	0.488
Ratio dogs to children	0·34 (0·256, 0·449)	0·27 (0·209, 0·354)	0.20	0.229
Proportion population literate	0·40 (0·356, 0·444)	0·48 (0·417, 0·545)	1.40	0.088
Proportion population employed	0·21 (0·189, 0·241)	0·27 (0·200, 0·258)	0.83	0.408
Proportion houses with dog	0·39 (0·300, 0·522)	0·35 (0·266, 0·467)	0.33	0.740
Village altitude (m)	664 (544·4, 810·2)	674 (594·7, 763·5)	0.46	0.591
Ratio dogs to human	0·09 (0·070, 0·118)	0·08 (0·059, 0·102)	1.09	0.275

In the region, 100–150 new VL cases were reported annually between 1998 and 2005, representing 45% of the total VL cases in Iran, the vast majority being children <10yrs of age[9–11, 38]. At the time of the study, pediatric *L*. *infantum* infection incidence measured by Direct Agglutination Test (DAT) seroconversion was 2·4% compared to 1.9% by Leishmanin skin test (LST) reaction [31]. Canine DAT seroconversion incidence was 7%[31], with reported regional seroprevalences of 11%-22% [31, 38, 39]. In the same locations, the canine to human seroprevalence ratio was 1.4: 1 (11% vs 8%)[31] compared to 3.1:1 (22% vs 7%) six years earlier in 1995[39]. Transmission is predominantly peridomestic, with human exposure being independent of age and sex, and associated with endophilic *Phlebotomus* sand fly vectors and infected dogs[39–43]. The risk of childhood *Leishmania* seropositivity is associated with dog ownership, village dog density (28/km^2^, 95% C.I.: 23.6–32.1), and the dog to human ratio[39]. Two species of sand fly, *Phlebotomus (Larroussius) perfiliewi transcaucasicus* and *Ph*. *(L*.) *kandelakii*, are likely vectors in this region, being seasonally active for 4 months (late June to October)[41, 44].

The VL control program hinges on the District Ministry of Public Health (DTARH) which is responsible for the District Health Centers (DHCs) in Kalaybar and Ahar. These centers coordinate activities of 12 provincial Rural Health Centers (RHCs), which are run by trained medical staff and health officers. Each RHC supervises about ten village health posts (*khaneh behdasht*) where resident Health Promoters (*behvarz*) are responsible for disease surveillance, control implementation, and facilitate suspect VL cases attendance at the RHCs. The RHCs provide free VL diagnostic testing and treatment services, and refer those needing more specialist hospitalization to the DHCs or district hospital. Clinical VL is a notifiable disease.

### Study design

#### Randomisation and treatment allocation

**Fig 1:**
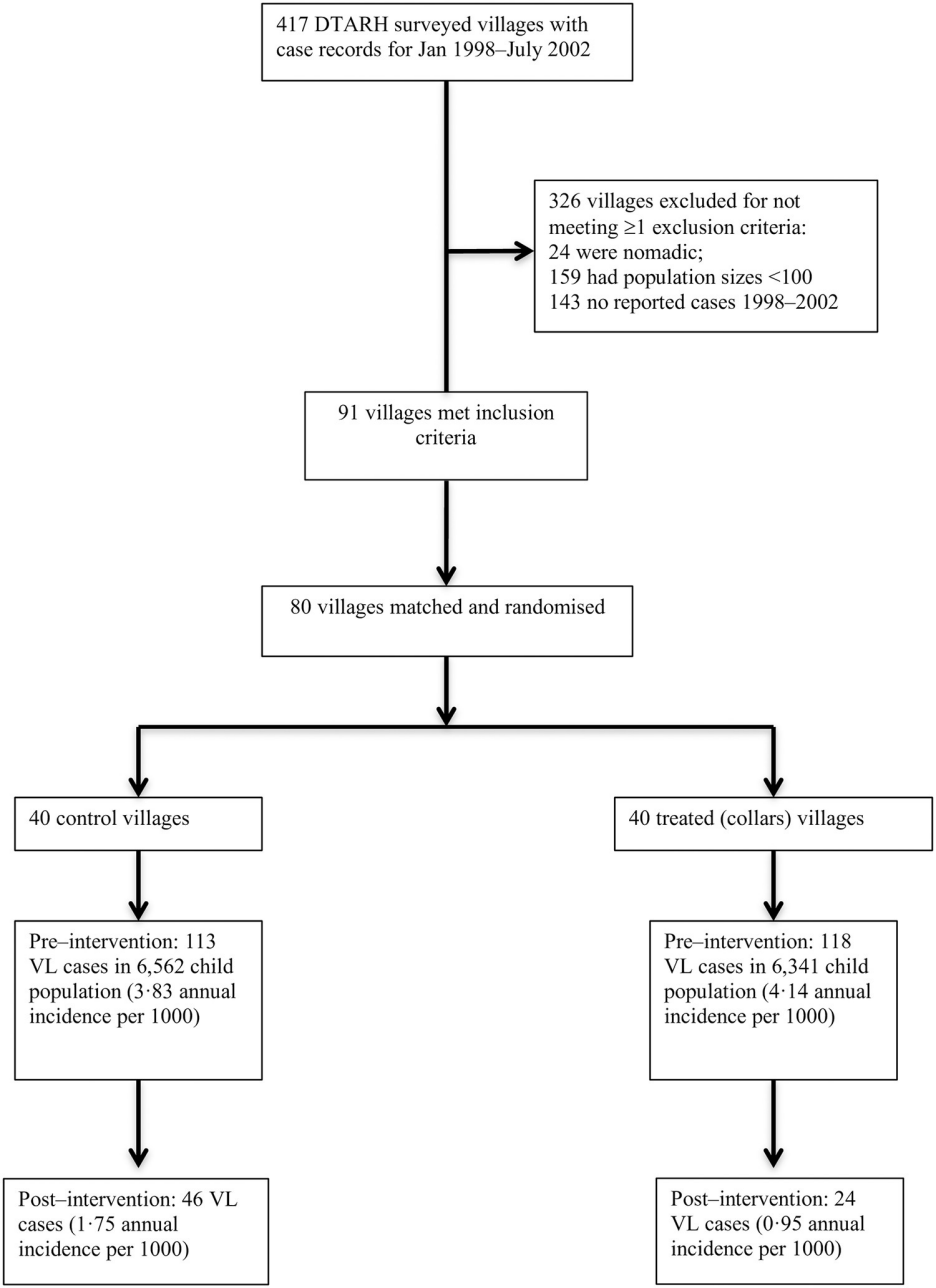
Trial design.

The trial was designed as a pair–matched cluster randomised trial[45]. Villages were designated as clusters and selected in a two–step procedure (Fig 1). All 417 endemic villages in the two districts, and confirmed VL case numbers in the 4·5 years prior to the intervention (January 1998– June 2002) were listed. The inclusion criteria for village recruitment were that the population size was >100, at least one confirmed clinical VL case was recorded in this period, and that the village population was not nomadic. A total of 91 villages met these criteria.

Following consultation by the RHC supervisors with village leaders and health workers, 80 of the more accessible villages were finally recruited. Ranked in descending order of pre–intervention VL incidence, the top two villages were paired and randomly assigned to the treatment (collars) or control (no collars) group by tossing a coin in the presence of observers. All subsequent pairs were similarly assigned alternately to either the treatment or control group, resulting in 40 control and 40 pair-matched villages.

The pre–intervention DTARH house–to–house survey indicated that the collar and control villages (clusters) were demographically well balanced (Table 1). Although Iran has a national *Leishmania* control program, during the trial period neither insecticide spraying, canine test–and–slaughter, nor VL health education programs were conducted in the enrolled communities. However, 27 stray dogs were removed by the authorities (11 and 16 dogs from five and seven control and collar villages, respectively).

#### The intervention

Dog collars impregnated with 40mg/g deltamethrin (Scalibor ProtectorBand, Intervet International, Boxmeer, Netherlands), were fitted to household dogs in the 40 treatment villages in late June/early July in each of four consecutive years (2002–2005). The Scalibor collar label claimed 5–6 months insecticidal activity[25, 26] which exceeded the annual transmission season.

The intervention was implemented by DTARH operational DHCs in Kalaybar and Ahar. The collars were donated by Intervet, and given to the DHCs who distributed them to the village Health Promoters, ensuring that sufficient collars and replacements were supplied each year. Health Promoters conduct routine house–to–house visitation forming part of the DTARH public health advisory and monitoring system. On these visits, they fitted the collars to dogs, promoted the correct use of the collars, recorded the daily numbers of collars fitted, lost, replaced, or refusals to fit collars (defined here as non-compliance), and identified any adverse clinical effects reported by dog owners. The authors helped train the Health Promoters to systematically record these events.

#### VL case definition and reporting

Confirmed clinical VL case numbers were extracted from secondary records provided by DTARH. At the time of the study, the DTARH protocol stated that suspect VL cases were considered confirmed if they presented clinical signs including prolonged fever, lymphadenopathy, and hepatosplenomegaly, associated with a positive DAT (threshold ≥1/3200 titer), and/or microscopic detection of *Leishmania* in clinical aspirates. All confirmed cases received treatment following WHO guidelines[12]. Based on parasite infections found in sand flies[42], *Leishmania tropica* is also transmitted in the region. However, this parasite typically causes cutaneous infections, does not normally induce DAT seroconversion, and the distinctive clinical signs are known by the DHCs.

#### Data management and masking

DTARH collated details of confirmed VL incident cases which were provided to the authors anonymised accompanied by records of the patient’s age, sex, village of residence, and date when they first presented at the medical center.

Lacking placebo collars, the village Health Promotors were not blinded to the intervention, but the clinicians and DTARH data managers who collated the case data were.

#### Trial outcomes

The primary outcome for analysis was the cumulative number of incident VL cases per village by the end of the 48 months follow–up period. The transmission season of infection was attributed based on the date of the patient’s initial presentation: dates falling between July_year ×_ and end of June_year ×+1_ were assigned to transmission season_year ×_. This interval allowed for a ≥9 month incubation period which exceeds the usual 2–6 months incubation period reported in Iran and elsewhere[38].

#### Sample size calculations

The study was statistically powered to detect a 55% reduction in the 48 month clinical VL incidence in collar clusters compared to control clusters, from baseline 0.004 (~3.978 cases per 1000 children ≤10yrs old/yr) calculated for the 4·5 yr pre–intervention period in the 80 trial clusters, and a surveyed harmonic mean of 106 children per cluster. Data from the previous collar trial in the region[31], gave an inter–cluster coefficient of variation of κ = 0·124 indicative of the degree of cluster pairwise matching achievable. We applied a more conservative κ = 0·25 in power calculations. Under these assumptions, 38 clusters per arm were required to achieve a power of 90% with α = 0·05 to reject the null hypothesis. The final number of clusters enrolled was 40 clusters per arm. Comparison of unpublished DTARH and published[39] demographic records showed that the village humans and dog populations were generally stationary and stable.

### Statistical analysis

The intervention effect was computed using random–effects Poisson regression to test differences in the cumulative numbers of incident VL cases per cluster (= village), expressed as an incidence risk ratio (IRR). The model included variables describing the trial design: the attributed year of infection, the log_10_ transformed childhood baseline village incidence, and the pair–matching structure (as a cluster term). The number of children ≤10yrs per village, being the well documented high risk group, was set as the model offset parameter. The variance in cluster ratios of children to total population size were similar between treatment arms (Table 1). Additional demographic variables, listed in Table 1, were then individually tested by log–likelihood ratio test [LRT] of nested models, as potential modifiers of the unadjusted intervention effect estimates.

A secondary analysis following[45] tested the normalized residuals of the observed /expected case ratios for pair-matched clusters (Supplementary S1), where the expected number of cases per cluster were generated first by fitting the Poisson model, simultaneously adjusting for significant covariates as described above, but excluding the intervention term. Then, normalized (square root transformed) residuals of the observed /expected case ratios were tested by Student’s paired *t*-test where the results of each cluster are given equal weighting[45]. Differences in these ratios were also confirmed by applying a non–parametric two–sided weighted signed rank test.

The relationship between post-intervention VL incidence differences between pair-matched villages, and pre-intervention incidence, was examined by linear regression (illustrated in Fig 4). The relationships between village incidence pre–and post–intervention was evaluated by negative binomial regression (nbreg), and by Spearman’s rank correlation (illustrated in Fig 5).

Data were analysed in Stata v.15.1 (StataCorp LP, College Station, TX).

### Ethical considerations

The trial protocol was approved by the Regional Committee of Medical Ethics, Tabriz University of Medical Sciences, Iran. The University of Warwick’s Biomedical and Scientific Research Ethics Committee (BSREC) confirmed that the trial raised no significant ethical concerns due to its use of secondary anonymised human case data. There was no local animal ethical committee at the time of the study. Informed written consent was obtained from village leaders, and from dog owners to fit collars, and dogs were monitored on a regular basis by village Health Promotors for any adverse reactions to the collars.

## Results

In the 4·5 years prior to the intervention (January 1998– June 2002), 113 confirmed clinical VL cases were reported in a population of 6,562 children in the 40 control villages, with an average annual case incidence of 3·83 per 1000. In the 40 collar villages during the same period, 118 cases were recorded amongst 6,341 children, an average incidence of 4·14 per 1000. These rates were not significantly different (IRR = 1·04 [95% C.L.s 0·802, 1·344], p>0·77).

**Fig 2:**
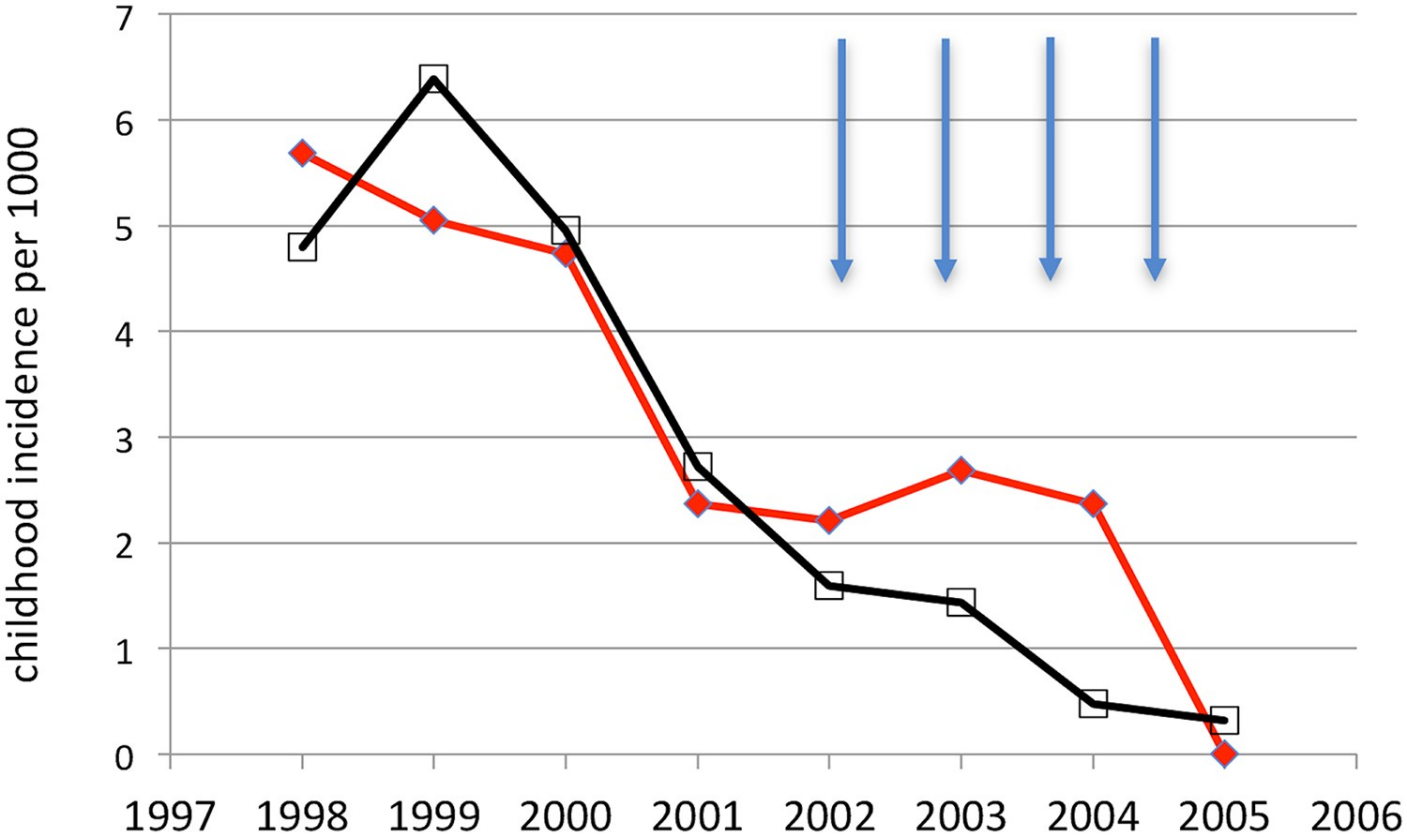
Confirmed VL case incidence during the 1998 to 2005 transmission seasons in collar villages (black line, N = 40) and control villages (red line, N = 40). Arrow represents when collars were fitted in late June/early July of each year before the start of transmission.

During the 48 month intervention period, 24 compared to 46 clinical VL cases were recorded in the collar and control arms, respectively. The corresponding incidences were 0·95/1000/yr and 1·75/1000/yr. Accounting for a general decline in VL case numbers over the years of observation in both trial arms (Fig 2), there was a significant cumulative protective effect attributed to the collar intervention (LR test: χ^2^_(1)_ = 7·83, p = 0·0051). The trial design–adjusted incidence risk ratio (IRR) was 0·50 (95% C.I. 0·300, 0·822) (model fit: Wald χ^2^_(5)_ = 25·7, p<0·0001), equivalent to 50% (95% C.I. 17·8%, 70·0%) protection against clinical VL.

The intervention effect was further confirmed by showing a significantly lower than expected number of cases in collared *vs* control villages (Student’s *t* = -3.39, df = 39, p = 0·0016) (Supplementary S1). The non–parametric sign rank test of these residuals was also significant (p = 0·008).

Additional demographic variables (listed in Table 1) were individually tested for effect estimate modification, but none of the variables significantly improved the model fit (LRT: χ^2^_(1)_ <1·92, p>0·187).

### VL case characteristics

The median age of the 70 reported clinical cases during the trial period was 1.6yrs (range: 0.5–5yrs), with M:F sex ratios of 25:21 and 12:12 in control and collar arms, respectively. There were no significant differences in case age distributions between trial arms (Poisson regression: z = -0·97, P = 0·331), nor between intervention years (non–parametric test for trend: z = -1·54, p = 0·124).

### Heterogeneities in the intervention effect

#### Year of intervention

The annual point estimates of the intervention effect suggested that the protective effect steadily increased with time under intervention, though the case numbers were low and the errors broad (Table 2).

**Table 2:** Crude and adjusted clinical VL incidence risk ratios (IRR), and observed number of confirmed VL cases, per year under intervention.

Year of intervention	IRR (95% CLs) adjusted for trial design characteristics^1^	IRR (95% CLs) unadjusted for trial design characteristics^2^	VL cases (number in collar/control arm)^3^
2002	0·69 (0·31, 1·57)	0·74 (0·33, 1·66)	10/14
2003	0·51 (0·23, 1·16)	0·55 (0·24, 1·23)	9/17
2004	0·19 (0·06, 0·67)	0·21 (0·06, 0·71)	3/15
2005	–	–	2/0
Average across 2002–2005	0·50 (0·30, 0·82)	0·54 (0·33, 0·88)	24/46

^1^ computed by fitting the RE Poisson model adjusted for trial design characteristics

^2^ computed from analysis of the raw annual risk ratios in absence of trial design characteristics

^3^ observed number of confirmed VL cases

#### Variation in the intervention effect between villages

By the end of the intervention, 12 (30%) of the 40 collar villages, compared to 24 (60%) of the 40 control villages, presented ≥1 clinical VL case, indicating a reduced risk also at the village level (IRR = 0·57 [95% C.I.s 0·372, 0·879], χ^2^_(1)_ = 7·27, p = 0·007).

**Fig 3:**
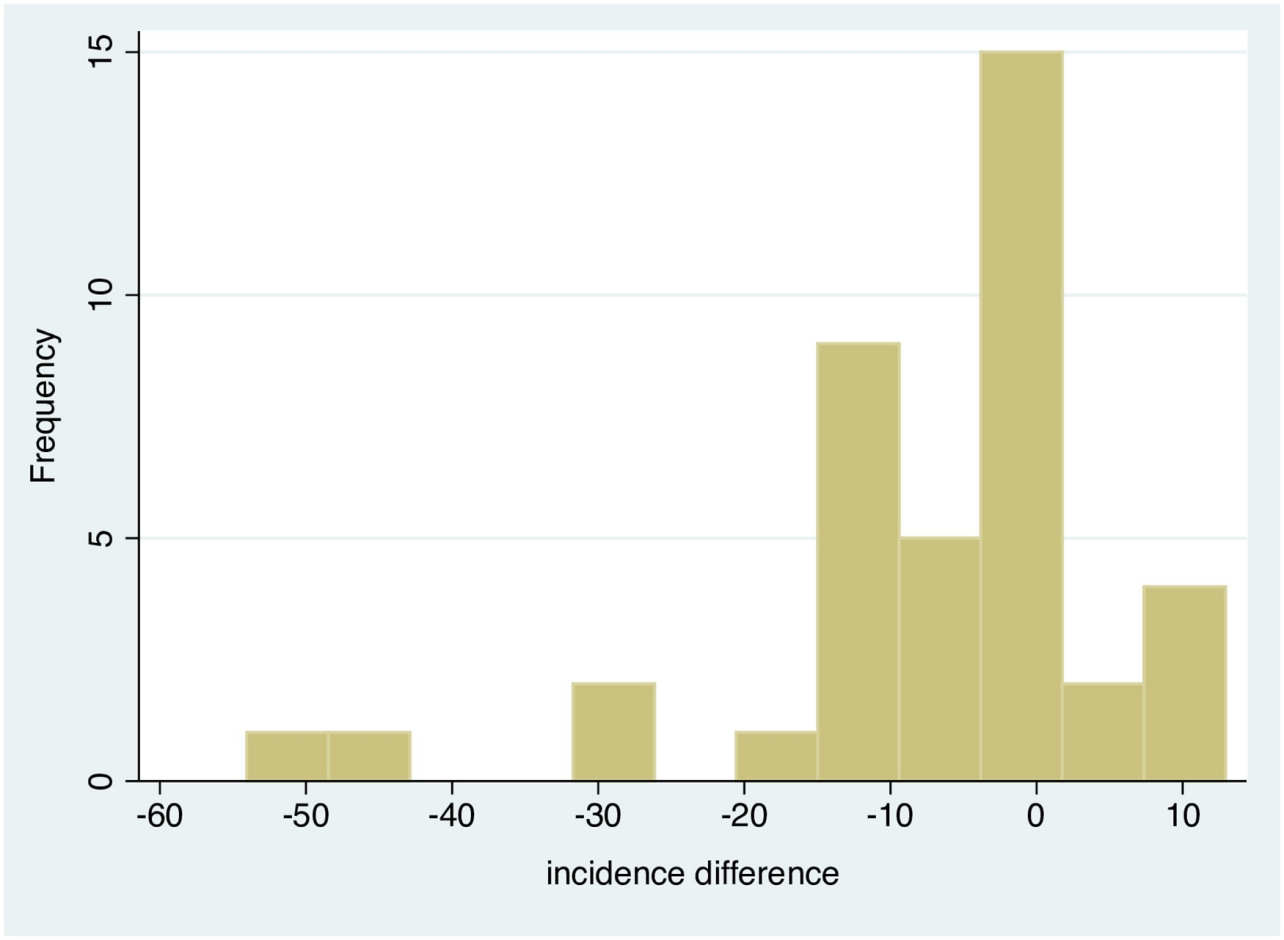
Frequency distribution of the absolute differences in post–intervention VL case incidence per 1000 between the 40 pair–matched collar and control villages. Negative differences represent a reduction in incidence attributed to the collar intervention. Positive changes represent an increase in incidence in the collar compared to the pair–matched control village. Values calculated as (post incidence collar–control) for pair–matched villages.

Eleven of the 40 paired–matched villages reported zero cases post–intervention in both treatment arms. Of the remaining 29 pairs, 22 (76%) collared villages showed reductions in incidence compared to the pair-matched control villages (Fig 3), with 11 new VL cases recorded compared to 42 VL cases in the control villages. However, in seven collared villages, there was an increase in incidence, with 13 VL cases compared to 4 cases in the pair-matched control villages over the 4 year period.

**Fig 4:**
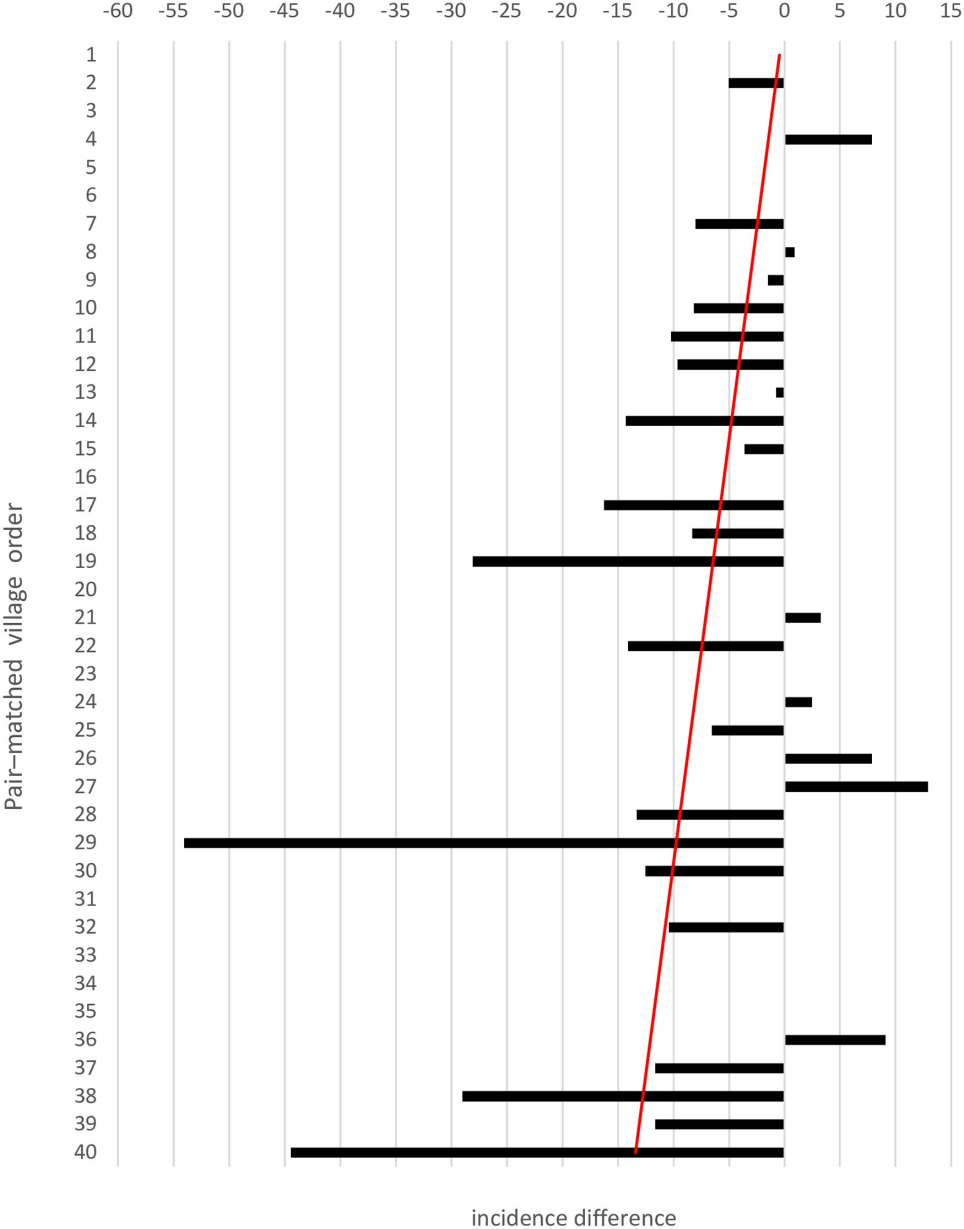
The absolute differences in the post–intervention period VL case incidence per 1000 between the pair–matched collar and control villages. Villages were pair–matched based on their pre–intervention incidence as described in the Methods, and ordered from low to high (Y axis: numbers 1–40). Dashed line: least squares linear fit from regression of the post–intervention incidence difference against the pre–intervention incidence.

The difference in post-intervention VL incidence between pair-matched villages attributed to the intervention was negatively associated with their pre-intervention baseline incidence (linear regression b = -0·427, z = -3·19, p<0.001) (Fig 4). This suggested that despite the general decline in VL case numbers by the end of the study, the intervention had a larger impact on higher compared to lower levels of pre-existing transmission intensity.

**Fig 5:**
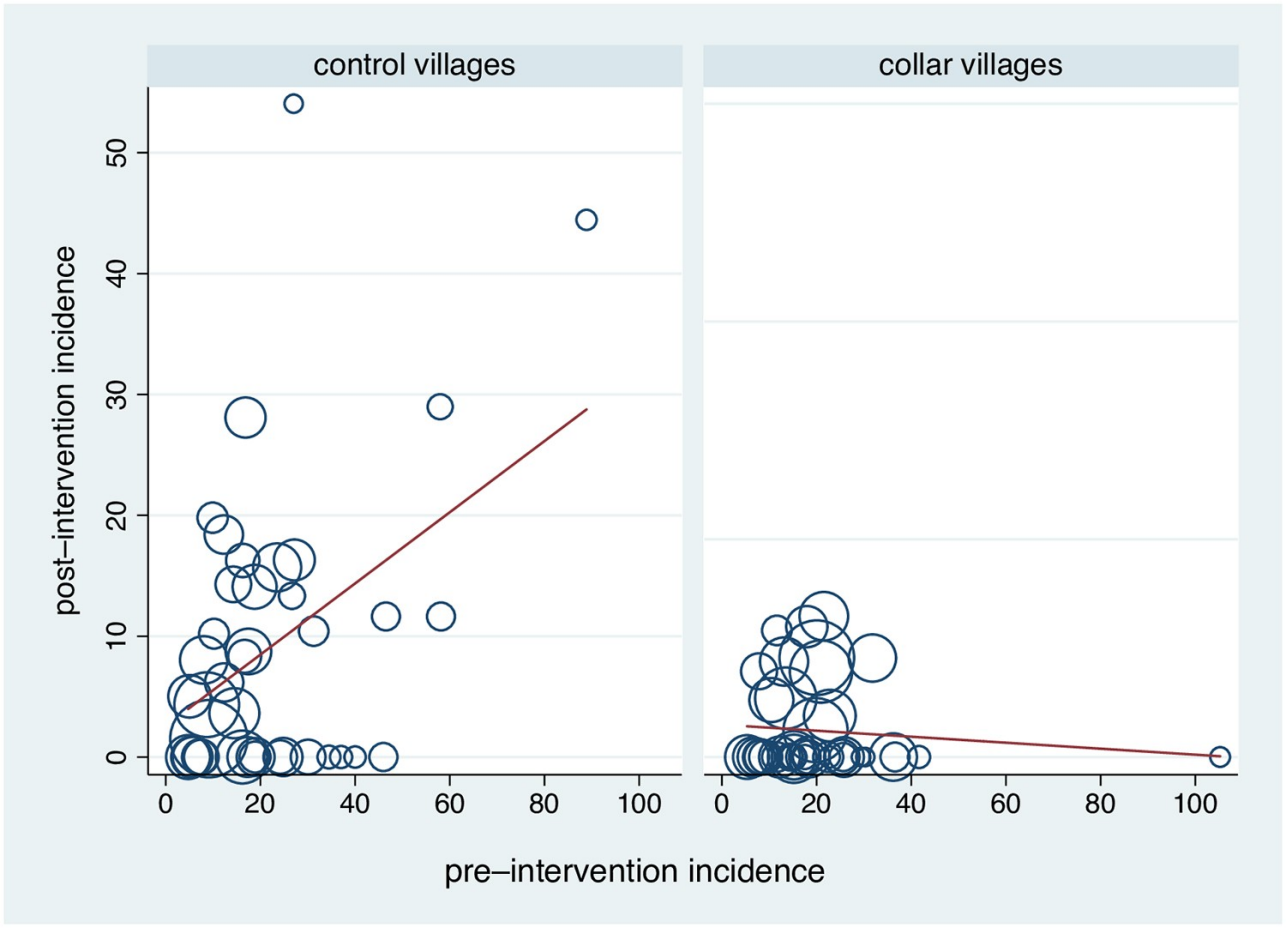
Association between the pre–and post–intervention incidence per 1000 in the 40 control and 40 collar villages. Line: least squares linear fit from regression of the post–intervention incidence against the pre–intervention incidence, weighted by number of children in the denominator (symbol size).

In the control arm, there was a positive association between village pre–and post–intervention incidence (nbreg: b = 0·095, z = 1·86, p = 0·06; Spearman’s r = 0·420, p = 0·007) (Fig 5). This relationship was interrupted by the intervention (nbreg: b = -0·107, z = -0·75, p = 0·45; Spearman’s r = -0·109, p = 0·50) (Fig 5).

### Collar coverage

**Fig 6:**
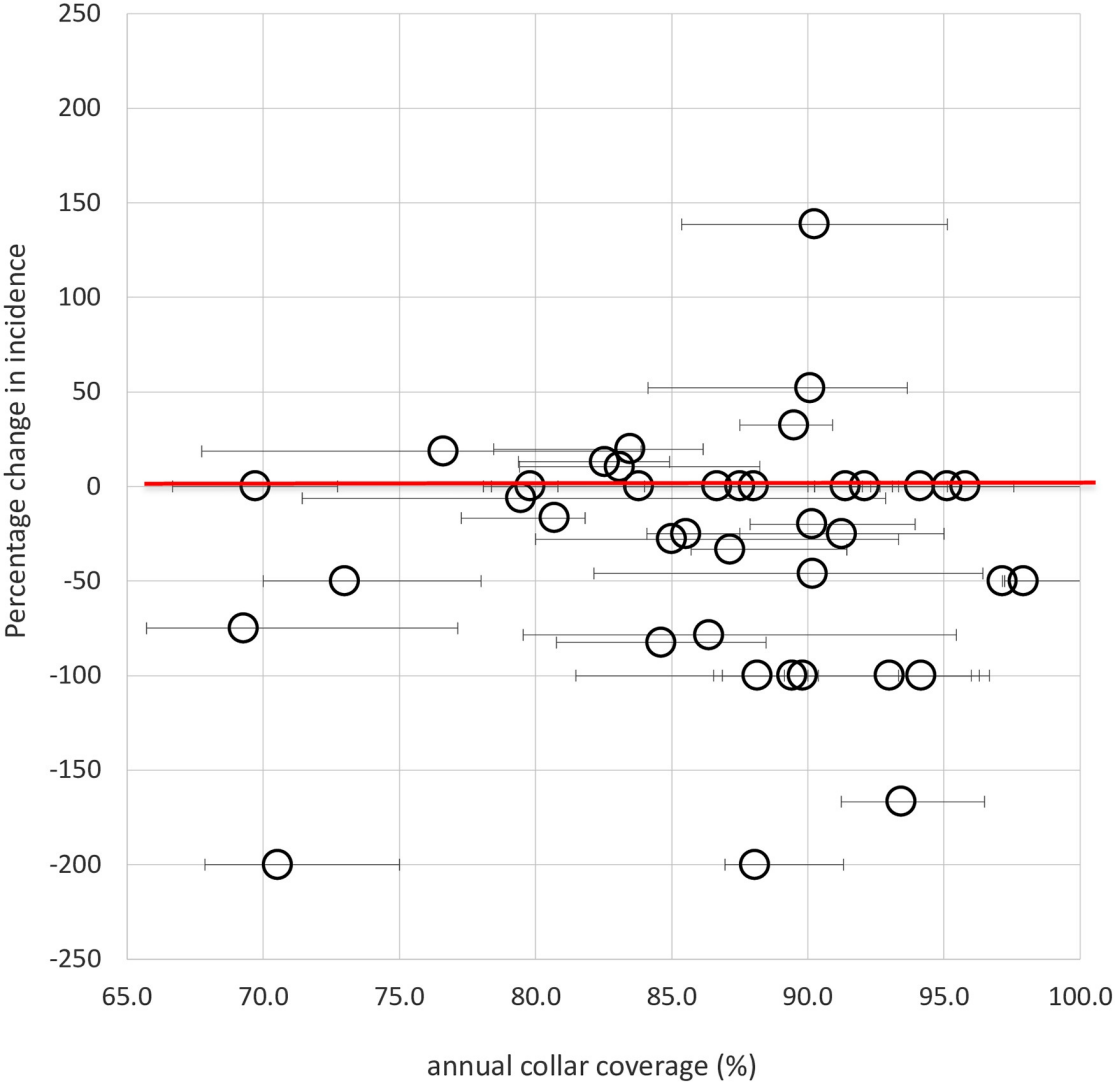
Association between the intervention effect on pair–matched collar and control village incidence per 1000 and the village annual collar coverage level (circles). Y axis error bars represent the minimum and maximum annual dog collar coverage per village over the 4 years intervention.

Over the trial period, 6,835 dogs were fitted with collars before the beginning of the sand fly season (1,682, 1,722, 1,689, and 1,742 dogs annually in the 4 years). Of these, 6.9% (95% C.I. 6.25%, 7.56%) were lost per year, respectively 7·3% (123/1,682), 6.4% (110/1722), 6.9% (117/1689) and 7.0% (122/1742). The mean annual coverage (percent of total dogs collared) was 87% (95% C.I. 84·2, 89·0%) being consistent across years (Kruskal–Wallis test: χ^2^_(3)_ = 0·260, p = 0·967), although it varied significantly between villages (range: 65·7%–100%) (Kruskal–Wallis test: χ^2^_(39)_ = 0·122, p = 0·0001) (Fig 6). Coverage in any single village and year was not associated with the village dog population size (z = 1·14, p = 0·26).

The variation in post-intervention VL incidence between pair-matched villages were not associated with the mean or variance in village collar coverage (t<0.67, p>0.51, r^2^ = 0.008); similar coverage was observed in villages with no incident VL cases, as those with >0 incident VL cases (Fig 6).

### Intervention logistics

#### Collar fitting, loss and replacement rates

Based on the most complete daily records (2002 data), collars were distributed by the DHCs to all village Health Promoters within 16 days at the start of the study. Aligned from the date (19^th^ June 2002) of supply to the first village, all collars were fitted in the 40 villages within one month.

In individual villages, all collars were fitted within a median 14 days (IQR 12.3–14.0; range: 4–30 days) from collar provision, with >70% in place within eight days. The number of dogs per village did not influence either the total fitting time (quantile regression: t = 0·95, p = 0·348) or daily fitting rates (comparison of daily slope interactions: z<0·71, p>0·54). Fitting rates were similar for guard and shepherd dogs (Two-sample Wilcoxon rank-sum test: z = -0.262, p = 0.794).

Collars were lost in 35 of the 40 collar villages, 2–147 days post-fitting. A median 3 (IQR 2–4; range 1–10) collars were lost per village. However, these were rapidly replaced: 59% within 4 days, and 91% within 8 days.

## Discussion

The effectiveness of insecticide–impregnated collars against human clinical VL had not been trialled as a potential public health control option. Community–wide collar coverage over 4 transmission seasons reduced the relative risk of infantile VL by 50% (95% C.I. 30%, 82%), and reduced the total VL case numbers by 48% during the intervention period.

The protective effect was clear despite a 70% general decline in reported case numbers over the study period, the decline being consistent with national trends from the late 1990s to 2014 in Iran[9] and elsewhere: waxing and waning cycles in incident case numbers are reported in the Indian subcontinent[5], with speculation over the regulating mechanisms[46]. In Iran, VL is a notifiable disease, subject of major public health programs, and well known to physicians in the study region. Development of VL from subclinical infection in the region is 8% (1 in 13 infections), where conversion risk declines with age associated with a rise in cell–mediated immunity[40]. The observed VL cases were ≤5yrs old, similar to the VL age–distributions throughout Iran[9–11]. This contrasts to the older average age groups afflicted with VL due to *L*. *donovani* infection[47], and in immunocompromised VL patients generally[16]. Traditionally a disease of children, variability in *L*. *infantum* VL case age distributions are appearing[23].

All eight published studies designed to evaluate the efficacy of Scalibor collars, demonstrated reduced canine seroinfection incidence by 46%–86% within 1–2 years of use[30–35, 37, 48]. They have been shown to reduce also sand fly densities[27], and sand fly infection prevalence with *L*. *infantum*[48]. The DTARH program did not monitor changes in canine infection rates or sand fly densities during the trial period, which is one limitation of this study. However, a previous cluster randomized trial of community-wide distribution of Scalibor collars in the study region, reported protective effects against canine infection of 54% [95% C.L.s 30%, 70%]), associated with a reduction in infection (*cf*. clinical disease) incidence of 43% (95% C.L.s 10%, 63%) in ≤10yr old children[31]. What was not clear from that study, was whether reductions in seroconversion would directly translate into reductions in clinical VL incidence. Scalibor collars release deltamethrin into dermal secretions, resulting in reduced sand fly blood–feeding by >90%, and increased mortality in blood–fed sand flies by 35%–100% over 8 months[25, 26]. More recent studies suggest that Scalibor collars offer 94–98% protection against *Phlebotomous perniciosus* sand fly bites for up to 12 months[49]. These effects reduce the likelihood of a collared dog acquiring infection and being a source for onward transmission, thus, they are expected to reduce the number of infectious bites on humans. The risk of clinical development is associated with elevated parasite burdens and immune responses[50, 51]; collars may also reduce the metacyclic inoculum delivered by vectors, and/or the initial amastigote infection burdens, sufficient to reduce the subclinical to clinical VL conversion rate. Lower incident anti–*Leishmania* antibody titers, observed in seronegative collared dogs[32], support this hypothesis.

The annual effect estimates in this study suggest that one to two years of continued intervention achieved a similar outcome to the 4-year cumulative effect. However, the point estimate errors were broad as the study was not statistically powered to detect significant changes in any single transmission season. Interpretation of the effect estimates over time was further hindered by the very low VL case numbers in both arms and inevitable stochastic events in the latter years of the study. A different cyclical pattern of VL between control and intervention villages is unlikely to have occurred due to the randomization of trial clusters.

Clinical VL cases continued to be notified in villages during the years of collar use, indicating that collars were not wholly effective in interrupting transmission. Villages with higher pre–intervention incidence were more likely to experience continued transmission, despite achieving greater relative reductions in disease incidence compared to villages of low pre–intervention incidence. This raises the possibility that the collar coverage was insufficient in some villages.

Collar coverage showed significant variation between villages with some being consistently low. Changes in village VL incidence was not associated with village coverage, nor indeed with any demographic variable. In three of the seven collared villages where the intervention failed to prevent a higher number of incident cases compared to controls, annual collar coverage was >84% (average c.90%); and in all except one of the 7 villages, the minimum annual coverage was >77%. Therefore, we do not attribute these failures to lower collar coverage levels. Nor do we attribute it to collar losses or to non–compliance (owner refusing to have collars fitted to dogs). Short–term allergic dermal reactions to deltamethrin, reported in 2%–16% of collared dogs in two Brazilian studies[32, 37], usually cause owners to remove the collars. Such adverse effects were not reported in this trial, nor in the previous trial in NW Iran[31]. Similarly, there were no cases of dog owners refusing to have collars fitted to their dogs by the Health Promoters.

Dogs ≤3m old were not collared (following label instructions) but represented <3% of the canine population. The period of latency to infectious onset far exceeds the 4 month transmission season[6], thus ruling out these dogs as significant reservoirs for continued transmission. Stray dogs were rarely seen (27 in total), equivalent to <0·2 dogs per study village year. Although local wild canid populations can support infections[38], their densities are relatively low, and the role of wild canids in maintaining transmission is disputed[8, 52, 53].

### Potential scale-up and sustainability

A significant finding of this study was that the intervention effect was achieved under the operational conditions as implemented by the DTARH VL control program. After ensuring that the design and village randomisation processes met CRT analytical requirements, the authors had minimal contact with authorities. The authors supplied known numbers of collars to the DHCs who distributed them to the villages, and collated details of the collar implementation. This provided the opportunity to measure the logistics and likely sustainability of programmatic scale–up.

Collars were fitted within only 15 days of village supply, and collar losses were rapidly replaced, suggesting coverage levels were maintained during the transmission seasons. Neither coverage level nor time to complete collar fitting were related to the total number of dogs eligible for a collar, concluding that lower village coverage levels were not due to time constraints. Feasibility of a regional–level scale–up was indicated by DTARH taking only 16 days to deliver collars to all 40 villages spread over an area of 15,000km^2^, and total collars being fitted within one month from collar provision to the first village.

Collars were socially accepted, visibly distinguished treated dogs, and should not require replacement every 3–4 weeks, unlike alternative topical formulations. Coupled with the short transmission season, effective collar duration, and rapid replacement rate, the feasibility of effective sustainable scale–up is indicated. Collar losses in this region of between 1% per month[31] and 1.7% (95% CI: 1.6%, 1.9%) per month (this study), were not substantially higher than in other endemic regions: 0·8% in Brazil[37] and 0·6–0·7% in Italy[34, 54], though exceptional rates, 7·8% and 8·2%, in both continents have been reported[32, 33].

Sustainability will be governed also by costs and cost–effectiveness. Considering the cost of collars alone, in this study, collars were donated by the manufacturer. However, based on €14 per collar, the annual material cost would have equated to approximately €24,000, or €96,000 over 4 years. That is €4,364 per VL case averted compared to case numbers in the control arm. This value is for a period with 70% relative decline in reported case numbers from the late 1990s to 2014[9]. Better cost–effectiveness may be expected as the cyclical waning in case numbers inevitably reverses in non–intervention regions.

One key knowledge gap is how best to implement dog collar programs: currently unknown is the minimum community coverage level necessary to reduce transmission, equivalent to vaccination coverage thresholds calculated to maintain herd immunity[55]. Mathematical models suggest that increasing collar coverage from 70% to 90% could cause moderate to substantial reductions in both canine and human infection prevalences[32, 56, 57]. However, three empirical studies that report coverage of 70%–100% failed to corroborate these predictions[31, 34, 37]. Without known target thresholds, collar campaigns may result only in protection of the individual dog or its household.

Of relevance to NTD elimination strategies, our data suggest that greater initial reductions in incidence may be achieved in communities with higher compared to lower preexisting transmission pressure. One prediction is that by reducing transmission, infection in the lower-prevalence settings becomes more clustered, thus more stable and more resilient to interventions[58]. Approaching elimination will require different control strategies to those used in the initial attack phase. For example, heterogeneities in household sand fly densities, and the transmission potential of individual vectors and reservoirs[53, 59, 60], imply the need for high coverage levels to encounter such transmission hotspots.

In conclusion, the results of this study promote the community-wide application of insecticide–impregnated collars as a public health tool to reduce clinical VL burdens, not just human and canine infection. The observed level of impact was achieved working within the existing MoH infrastructure, demonstrating many desirable attributes of a potentially sustainable control program that could be scaled–up with minimal additional technical capacity training.

## Supporting information

S1 Table Post–intervention observed and expected case incidence of infantile VL by the end of the four year intervention period. (PDF)
