# Supplementary material for: Insecticide–impregnated dog collars reduce infantile clinical visceral leishmaniasis under operational conditions in NW Iran: A community–wide cluster randomised trial
Source: PLoS Negl Trop Dis. 2019 Mar 4;13(3):e0007193. doi: 10.1371/journal.pntd.0007193 (PMC6417739; doi:10.1371/journal.pntd.0007193)
Supplement: S1 Table — (PDF) [file pntd.0007193.s001.pdf]

Supplementary Information

S1. Post–intervention observed and expected case incidence of VL by the end of the four year intervention period.

Control village	Observed % (cases/child population)	Expected childhood cases %	O/E ratio
1	0·00% (0/212)	0·27	0·00
2	0·50% (1/199)	0·27	1·83
3	0·00% (0/183)	0·28	0·00
4	0·00% (0/176)	0·66	0·00
5	0·00% (0/139)	0·32	0·00
6	0·00% (0/139)	0·34	0·00
7	0·80% (2/249)	0·39	2·07
8	0·43% (2/462)	0·43	1·01
9	0·15% (1/659)	0·43	0·35
10	1·98% (2/101)	0·38	5·24
11	1·02% (1/98)	1·09	0·93
12	1·84% (3/163)	0·42	4·35
13	0·62% (1/162)	0·46	1·34
14	1·43% (2/140)	0·45	3·20
15	0·36% (1/276)	0·46	0·78
16	0·00% (0/309)	0·49	0·00
17	1·63% (2/123)	0·48	3·40
18	0·83% (1/120)	0·49	1·71
19	2·81% (5/178)	0·50	5·66
20	0·00% (0/174)	0·52	0·00
21	0·87% (2/229)	0·53	1·65
22	1·41% (3/213)	0·56	2·52
23	0·00% (0/106)	0·56	0·00
24	0·00% (0/158)	0·52	0·00
25	1·57% (4/255)	0·65	2·42
26	0·00% (0/124)	0·59	0·00
27	0·00% (0/161)	0·63	0·00
28	1·33% (1/75)	0·66	2·03
29	5·41% (2/37)	0·57	9·49
30	1·63% (3/184)	0·70	2·32
31	0·00% (0/133)	0·73	0·00
32	1·04% (1/96)	0·64	1·62
33	0·00% (0/58)	0·68	0·00
34	0·00% (0/54)	0·87	0·00
35	0·00% (0/50)	0·86	0·00

36	0·00% (0/87)	0·90	0·00
37	1·16% (1/86)	0·86	1·35
38	2·90% (2/69)	1·00	2·91
39	1·16% (1/86)	0·97	1·20
40	4·44% (2/45)	1·23	3·62
Mean	0·70% (46/6562)	0·60	1·58

Collar villages	Observed % (cases/child population)	Expected childhood cases %	O/E ratio
1	0·00% (0/188)	0·27	0·00
2	0·00% (0/153)	0·31	0·00
3	0·00% (0/137)	0·33	0·00
4	0·79% (1/127)	0·34	2·32
5	0·00% (0/122)	0·37	0·00
6	0·00% (0/118)	0·39	0·00
7	0·00% (0/108)	0·37	0·00
8	0·52% (1/191)	0·40	1·32
9	0·00% (0/86)	0·39	0·00
10	1·16% (1/86)	0·43	2·68
11	0·00% (0/164)	0·43	0·00
12	0·88% (2/228)	0·48	1·84
13	0·54% (2/370)	0·50	1·08
14	0·00% (0/70)	0·41	0·00
15	0·00% (0/67)	0·47	0·00
16	0·00% (0/66)	0·47	0·00
17	0·00% (0/263)	0·51	0·00
18	0·00% (0/197)	0·51	0·00
19	0·00% (0/116)	0·53	0·00
20	0·00% (0/57)	0·58	0·00
21	1·20% (2/167)	0·56	2·15
22	0·00% (0/109)	0·58	0·00
23	0·00% (0/162)	0·55	0·00
24	0·25% (1/404)	0·60	0·41
25	0·91% (5/548)	0·68	1·34
26	0·79% (3/382)	0·63	1·24
27	1·29% (3/232)	0·67	1·93
28	0·00% (0/89)	0·61	0·00
29	0·00% (0/44)	0·59	0·00
30	0·38% (1/263)	0·63	0·60
31	0·00% (0/118)	1·01	0·00
32	0·00% (0/78)	0·62	0·00
33	0·00% (0/155)	0·65	0·00

34	0·00% (0/34)	0·77	0·00
35	0·00% (0/33)	0·74	0·00
36	0·91% (2/220)	0·74	1·23
37	0·00% (0/221)	0·80	0·00
38	0·00% (0/82)	0·81	0·00
39	0·00% (0/48)	0·79	0·00
40	0·00% (0/38)	1·05	0·00
Mean	0·38% (24/6341)	0·56	0·45
